# Suppressive effects of vitamin C-treated induced-regulatory T cells on heart allograft rejection under vitamin C-deficient or –sufficient conditions

**DOI:** 10.1371/journal.pone.0246967

**Published:** 2021-02-12

**Authors:** Ju Hee Hwang, Honglin Piao, Joon Young Jang, Sun-Kyung Lee, Dongkyu Han, Gwang-Min Lee, Cheolhyeon Go, Yejin Kim, Kwon Ik Oh, Jae Seung Kang, Ji-Jing Yan, Jaeseok Yang

**Affiliations:** 1 Biomedical Research Institute, Seoul National University Hospital, Seoul, Republic of Korea; 2 Department of Medicine, Graduate School, Seoul National University College of Medicine, Seoul, Republic of Korea; 3 Department of Anatomy and Cell Biology, Seoul National University College of Medicine, Seoul, Republic of Korea; 4 Institute of Allergy and Clinical Immunology, Seoul National University Medical Research Center, Seoul, Republic of Korea; 5 Department of Pathology, Hallym University College of Medicine, Chuncheon, Republic of Korea; 6 Transplantation Center, Seoul National University Hospital, Seoul, Republic of Korea; 7 Department of surgery, Seoul National University hospital, Seoul, Republic of Korea; Children’s Hospital Boston, UNITED STATES

## Abstract

Foxp3 stability of vitamin C-treated induced-regulatory T cells (V-iTregs) is superior to that of conventional iTregs (C-iTregs). However, the role of V-iTregs in allograft rejection under vitamin C-deficient conditions, such as those seen in humans, remains unclear. We aimed to elucidate the role of vitamin C treatment on generation and maintenance of iTregs from gulo knockout (Gulo-KO) mice as well as wild type (WT) mice, and *in vitro* and *in vivo* suppressive effects of V-iTregs on heart allograft rejection in either Gulo-KO or WT recipient mice. Conversion efficiency of iTregs was similar between C- and V-iTregs in both WT and Gulo-KO mice. V-iTregs from WT or Gulo-KO mice showed better *in vitro* Foxp3 stability than C-iTregs, although there was no difference between WT V-iTregs and Gulo-KO V-iTregs. Furthermore, V-iTregs from WT or Gulo-KO mice suppressed *in vitro* T cell proliferation better than C-iTregs. Heterotrophic heart transplantation from BALB/c mice to WT or vitamin C-deficient Gulo-KO C57BL/6J mice was performed following adoptive transfer of C- or V-iTregs. V-iTregs as well as C-iTregs prolonged heart allograft survival in WT and Gulo-KO mice. However, there was no difference between the C- and V-iTreg groups. Supplementation of low- or high-dose vitamin C did not induce significant changes in heart allograft survival in Gulo-KO recipients that had received V-iTregs. In conclusion, V-iTregs do not exert better suppressive effects on heart allograft survival than C-iTregs in either WT or vitamin C-deficient recipients.

## Introduction

Regulatory T cells (Tregs) play an important role in the induction and maintenance of transplantation tolerance as well as maintenance of self-tolerance, and infusion of Tregs has been shown to suppress allograft rejection [1–3]. However, the frequency of naturally occurring (nTregs) or thymic-derived Tregs (tTregs) is very low, requiring *in vitro* expansion of Tregs for effective cell therapy, which poses a risk of contaminated effector T cell outgrowth. Forkhead Box P3 (Foxp3) is essential for the development and functions of Tregs, especially nTregs or tTregs [4,5].

Non-regulatory T cells can be converted to Tregs *in vitro* under specific conditions, such as T cell receptor stimulation along with interleukin-2 (IL-2) and transforming growth factor-β (TGF-β) [6–9]. The benefit of these induced Tregs (iTregs) is that much higher yields can be achieved compared with nTreg. However, following conversion, these iTregs may lose Foxp3 expression, which is the factor that determines Treg lineage as well as their suppressive function [7,8,10–12]. Such instability of iTregs could be attributed to epigenetic modification in the Foxp3 region and one of well-known mechanisms is increased methylation in the CNS2 region within the Treg-specific demethylated region (TSDR) region of the Foxp3 gene [13,14]. As recently evidenced, vitamin C maintained demethylation at the CNS2 region of Foxp3 in a Tet-dependent manner, where vitamin C-treated iTregs (V-iTregs) maintained Foxp3 expression more stably than vitamin C-untreated iTregs, used a control (C-iTregs) [15–17]. Furthermore, these vitamin C-treated iTregs were found to suppress graft versus host disease more effectively than C-iTregs [18]. In addition, iTregs induced by a combination of vitamin C and vitamin A suppressed skin allograft rejection to a greater extent than C-iTregs did [19]. Nevertheless, the superiority of the suppressive effects exerted by V-iTregs on vascularized allograft rejection, over those exerted by C-iTregs remains unproven.

Humans cannot produce vitamin C by themselves as they lack L-gulonolactone-γ-oxidase (gulo), a rate-limiting enzyme involved in vitamin C synthesis, and therefore require vitamin C supplementation [20]. In contrast, mice carry intact gulo and produce vitamin C sufficiently. To study the role of vitamin C in humans, gulo knockout (Gulo-KO) mouse models lacking vitamin C have been used as an equivalent to humans [21]. The current study investigated the role of vitamin C treatment on generation and maintenance of iTregs from Gulo-KO mice as well as wild type (WT) mice, and *in vitro* and *in vivo* suppressive effects of V-iTregs on heart allograft rejection in either Gulo-KO or WT recipient mice. Furthermore, we investigated whether low-dose or high-dose oral vitamin C supplementation is beneficial in the immunosuppressive effects of V-iTregs on heart allograft rejection.

## Materials and methods

### Ethics statement

All animal use protocols were approved by the Institutional Animal Care and Use Committee of Seoul National University (SNU-180227-2-2, SNU-180704-3-2). All animals received humane care in compliance with the Principles of Laboratory Animal Care as formulated by the National Society for Medical Research.

### Animals

BALB/c, CD45.1^+^ and CD45.2^+^ C57BL/6J (B6) mice were purchased from Jackson Laboratory (Ellsworth, ME, USA). B6-Foxp3^GFP^ knockin (KI) mice were generously provided by A.Y. Rudensky (Memorial Sloan Kettering Cancer Center, New York, NY, USA). Gulo knockout (KO) mice were maintained by supplementing vitamin C (0.33g/L, sodium L-ascorbate, Sigma-Aldrich, St. Louis, MO, USA) in drinking water, and used for vitamin C-deficient conditions after discontinuation of vitamin C supplement for 3 weeks.

### Treg conversion and assessment of Foxp3 stability

Naïve splenic CD4^+^ T cells (CD4^+^CD25^-^CD44^low^ or CD4^+^Foxp3^-^CD44^low^ cells) were isolated from WT or Foxp3^GFP^ KI B6 mice by sorting via FACS Aria II (BD Biosciences, San Diego, CA, USA). To convert naïve CD4^+^ T cells to iTregs, sorted naïve CD4^+^ T cells were stimulated by anti-CD3 (1 μg/mL, clone 17A2, BioLgend, San Diego, CA, USA), anti-CD28 (1 μg/mL, clone 37.51, BioLgend), TGF-β (10 μg/mL, Peprotech, Rocky Hill, NJ, USA) and IL-2 (30 ng/mL, Thermo Fisher Scientific, Waltham, MA, USA) in the presence or absence of vitamin C (10 μg/mL, Ascorbic Acid, Sigma-Aldrich) for 5 d, according to a previous protocol [15]. To assess *in vitro* Foxp3 stability, iTregs were re-stimulated with anti-CD3, anti-CD28 (1–2 μg/mL, clone 37.51, BioLgend), and IL-2 with or without vitamin C and cultured for 2 weeks. In order to assess *in vivo* Foxp3 stability, CD45.1^+^ B6 iTreg (2 × 10^6^) were intravenously transferred to CD45.2^+^ WT or Gulo-KO B6 mice. Foxp3 expression in transferred iTregs was analyzed at 1 and 2 weeks via flow cytometry.

### *In vitro* suppression assay

Splenic CD4^+^ responder T cells were isolated from WT or Gulo-KO B6 mice using the MojoSort^TM^ Mouse CD4 T cell Isolation Kit (BioLegend), and were labeled with CTV (CellTrace^TM^ Violet Cell Proliferation Kit, Thermo Fisher Scientific). The Labeled T cells were co-cultured with iTregs at ratio of 2:1 and stimulated by anti-CD3 and anti-CD28 for 3 d. T cell proliferation was analyzed via flow cytometry, and results were expressed as a division index [22].

### Heart transplantation

Hearts from BALB/c mice were transplanted into the abdomen of WT or Gulo-KO B6 mice (S1 Appendix) [23]. One day prior to transplantation, iTreg cells from CD45.1^+^ Foxp3^GFP^ KI B6 mice (2 × 10^6^) were intravenously transferred into CD45.2^+^ recipient mice. Mice were anesthetized by inhalation of 1–2% of isoflurane (Hana Pharm, Republic of Korea). Tramadol (0.25 mg/kg; Jeil Pharmaceutical, Seoul, Republic of Korea) was subcutaneously administrated immediately after surgery to relieve pain. The recipient mice were cared in incubator chamber (Jeung Do Bio & Plant Co, Republic of Korea) at 27°C for a day to maintain body temperature. A palpation score of 0 in two consecutive tests was regarded as indicative of heart allograft rejection. The mice were euthanized upon heart allograft rejection by CO_2_ inhalation_._ In vitamin C supplementation experiments using Gulo-KO mice, low- or high-dose vitamin C was administered via drinking water bottles at a concentration of 0.33 g/L or 3.3 g/L, respectively [21].

### Flow cytometric analysis

Heart grafts and spleens were homogenized and were stained with the following antibodies: anti-CD8a (53–6.7), anti-CD44 (IM7), anti-CD25 (PC61), anti-CD11c (N418), anti-B220 (RA3-6B2) (BD Biosciences), anti-F4/80 (BM8), anti-Foxp3 (FJK-16S), anti-CD4 (GK1.5) (Thermo Fisher Scientific), anti-CD45 (30-F11), anti-CD3 (17A2), anti-CD45.1 (A20), and anti-CD45.2 (clone:104) (BioLegend) (S1 Table). 7-Aminoactinomycin D (7-AAD; BD Biosciences) was added to stain dead cells. Flow cytometry was performed using an Attune NxT Flow Cytometer (Thermo Fisher Scientific). Data were analyzed using FlowJo software (Tree Star, Ashland, OR, USA).

### Histological analysis

Heart allografts were fixed with 4% paraformaldehyde and their paraffin-embedded sections were stained using H & E (hematoxylin and eosin) stain. Tissue injury by rejection was semiquantitatively assessed according to a previous study [24]. Immunohistochemical staining was also performed using rabbit anti-CD3 (DAKO, Glostrup, Denmark) or anti-mouse F4/80 (clone BM8, Thermo Fisher Scientific), followed by a Dako Real Envision HRP kit (DAKO). The stained slides were assessed using an Olympus BX40 light microscope (Olympus, Japan), and images were analyzed with Image-Pro 5.1 software (Media Cybernetics, Rockville, MD, USA). All histological analyses were performed by two independent researchers blinded to the treatment group.

### Measurement of vitamin C concentration

Serum was mixed with an equal volume of 10% meta-phosphoric acid (Sigma-Aldrich) in high-performance liquid chromatography (HPLC) water (J.T. Baker, Philipsburg, NJ, USA) and reacted on a rotator at 4°C for 30 min. Following centrifugation, the supernatant was loaded onto a column (Dionex Ultimate 3000; Thermo Fisher Scientific) for HPLC analysis. Resulting analytes were separated on a Hypersil Gold C18 column (150 mm × 4.6 mm, 5 μm, ThermoFisher Scientific). The mobile phase consisted of water containing 0.1% acetic acid (J.T. Baker) (A) and methanol (J.T. Baker) (B). Elution proceeded using a 95% A solution and 5% B solution for 15 min at a flow rate of 1 mL/min.

### Statistical analysis

Data are shown as mean ± standard error of the mean (SEM) and analyzed via a two-tailed Student’s *t*-test or one-way analysis of variance (ANOVA) followed by Turkey’s post-hoc analysis. Graft survival was analyzed using the log-rank test. P < 0.05 was considered statistically significant. All analyses were performed using GraphPad Prism (v. 7.0; GraphPad Software, La Jolla, CA, USA).

## Results

### Impact of vitamin C on iTreg generation

**Fig 1:**
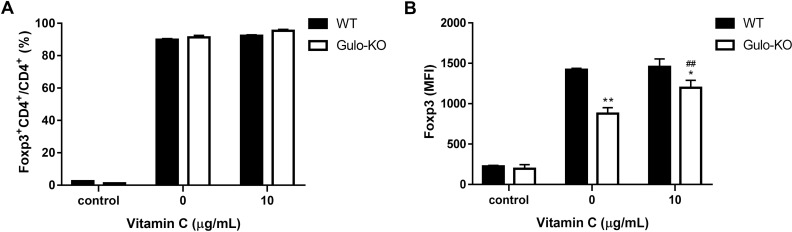
Impact of vitamin C on conversion to iTregs. (A) Proportions of Foxp3^+^ following *in vitro* conversion in C- and V-iTregs from either WT or Gulo-KO mice. (B) MFI of Foxp3 expression in C- and V-iTregs from either WT or Gulo-KO mice. Controls consists of unconverted T cells. N = 3 per group. Line and whiskers in the dot plots indicate the mean and standard error of the mean. *P < 0.05, **P < 0.01 for WT mice group vs. Gulo-KO mice group; ^##^P < 0.01 for no vitamin C group vs. 10 μg/mL vitamin C group by Student’s *t*-test. Foxp3, Forkhead Box P3; Gulo-KO, L-gulonolactone-γ-oxidase knockout; MFI, Mean fluorescent intensity; WT, wild type.

The proportion of Foxp3^+^ iTregs following *in vitro* conversion from naïve non-Tregs were similar between C- and V-iTregs in both WT and Gulo-KO mice (Fig 1A). However, the mean fluorescence intensity (MFI) of Foxp3 expression in iTregs from Gulo-KO mice was significantly lower than that in iTregs from WT mice (Fig 1B). Moreover, Foxp3 MFI in V-iTregs was also higher than that in C-iTregs in Gulo-KO mice (Fig 1B). When the proportions of Foxp3^+^CD4^+^ nTregs among CD4^+^ T cells (S1A Fig) and Foxp3 MFI of nTregs (S1B Fig) in the Gulo-KO mice were compared with those in the WT mice, there was no significant difference. Overall, vitamin C treatment did not have a significant impact on the conversion efficiency of iTregs in either WT or Gulo-KO mice. However, it increased Foxp3 MFI of iTregs in Gulo-KO mice.

### Impact of vitamin C on the *in vitro* stability of iTregs

**Fig 2:**
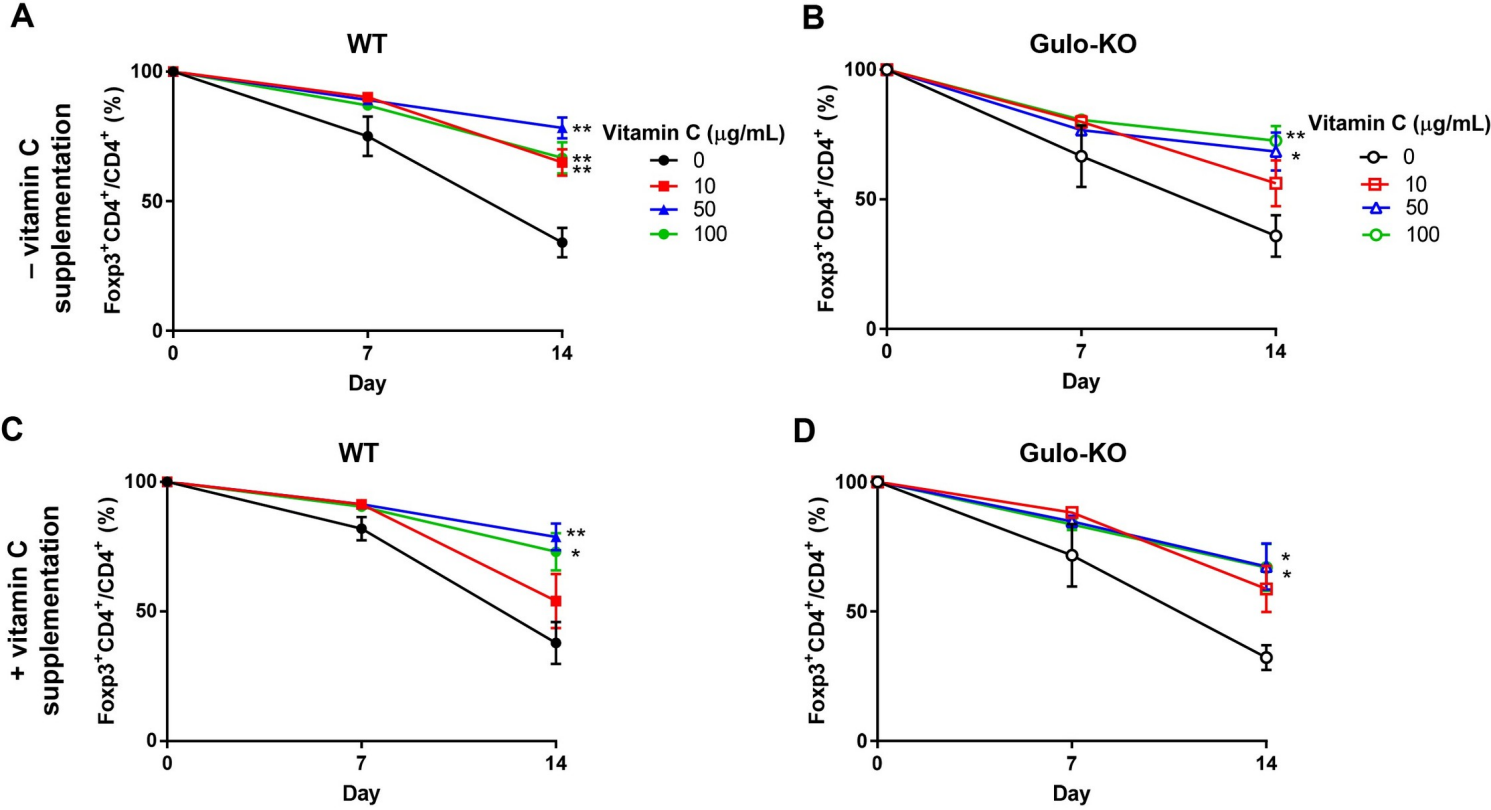
Impact of vitamin C on *in vitro* stability of iTregs. (A-B) V-iTregs from WT (A) or Gulo-KO (B) mice were induced in the presence of various concentration of vitamin C (10, 50, or 100 μg/mL). Next, these V-iTregs as well as C-iTregs from WT (A) or Gulo-KO (B) mice were cultured for 2 weeks in the absence of vitamin C. (C-D) V-iTregs from WT (C) or Gulo-KO (D) mice were induced in the presence of various concentrations of vitamin C (10, 50, or 100 μg/mL). V-iTregs and C-iTregs from WT (C) or Gulo-KO (D) were then cultured for 2 weeks in the presence of vitamin C (10 μg/mL). Proportions of Foxp3^+^ iTregs were measured after 1 and 2 weeks of culture. N = 9–12 per group. Line and whiskers in the dot plots indicate the mean and standard error of the mean. *P < 0.05, **P < 0.01 for C-iTreg group vs. V-iTreg group on day 14 by ANOVA. C-iTregs, control induced regulatory T cells without vitamin C treatment; Foxp3, Forkhead Box P3; Gulo-KO, L-gulonolactone-γ-oxidase knockout; V-iTregs, vitamin C-treated, induced regulatory T cells; WT, wild type.

V-iTregs from WT mice maintained Foxp3 without further vitamin C supplementation for 2 weeks after conversion to iTregs, whereas C-iTregs from WT mice lost Foxp3 expression over time (Fig 2A). Similarly, V-iTregs from Gulo-KO mice showed better *in vitro* stability of Foxp3 expression than C-iTregs (Fig 2B). Next, we investigated whether further vitamin C supplementation would improve Foxp3 maintenance in iTregs. Vitamin C supplementation did not show a significant impact on Foxp3 expression in either V-iTregs or C-iTregs from WT mice (Fig 2C). There was also no significant improvement in Foxp3 expression in either V-iTregs or C-iTregs from Gulo-KO mice (Fig 2D). Taken together, vitamin C supplementation during conversion improved Foxp3 stability of iTregs, whereas vitamin C supplementation after conversion did not contribute to the *in vitro* stability of Tregs.

### Impact of vitamin C on the *in vitro* suppressive activity of iTregs

**Fig 3:**
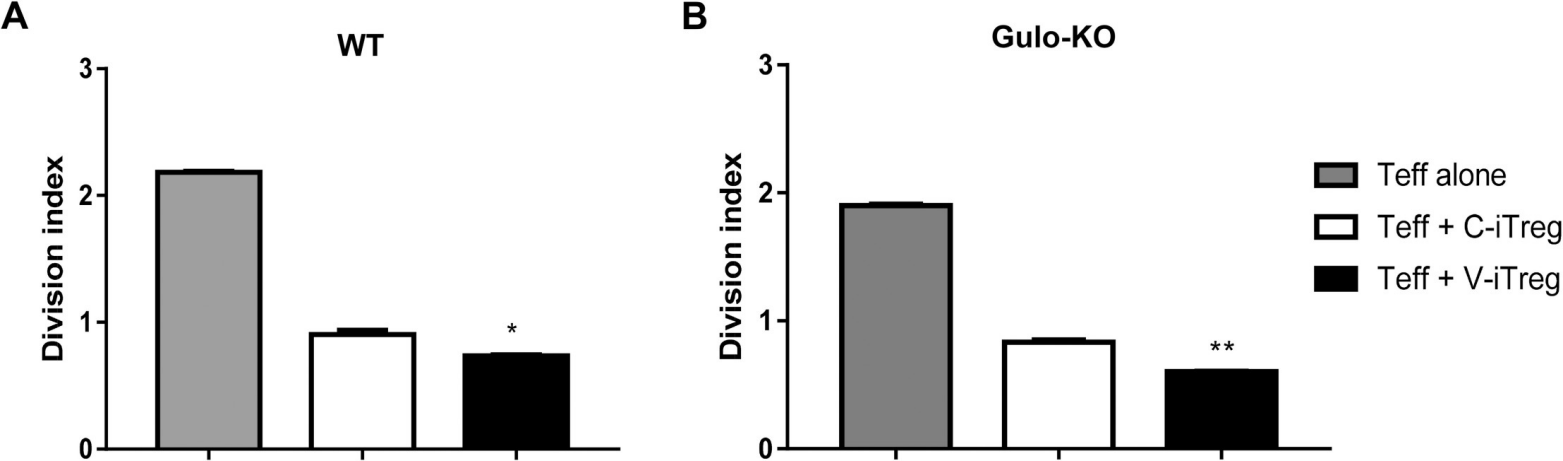
Impact of vitamin C on *in vitro* suppressive activity of iTregs. CD4^+^ T cells from WT mice were co-cultured with C-iTregs or V-iTregs from WT mice at a ratio of 1:1 or 2:1 and stimulated by anti-CD3 and anti-CD28 for 3 d (A). Similarly, CD4^+^ T cells from Gulo-KO mice were co-cultured with C-iTregs or V-iTregs from Gulo-KO mice (B). N = 3 per group. Line and whiskers in the dot plots indicate the mean and standard error of the mean. *P < 0.05, **P < 0.01 for C-iTreg group vs. V-iTreg group by Student’s *t*-test. C-iTregs, control induced regulatory T cells without vitamin C treatment; Foxp3, Forkhead Box P3; Gulo-KO, L-gulonolactone-γ-oxidase knockout; V-iTregs, vitamin C-treated, induced regulatory T cells; WT, wild type.

V-iTregs suppressed *in vitro* proliferation of T cells to a greater extent than C-iTregs in WT mice (Fig 3A). V-iTregs from Gulo-KO mice also showed slightly better *in vitro* suppressive activity against T cell proliferation than the V-iTregs from Gulo-KO mice (Fig 3B). These data indicate that V-iTregs displayed better *in vitro* suppressive activity than V-iTregs in both WT and Gulo-KO mice, although the difference was small.

### Impact of vitamin C on the *in vivo* suppressive activity of iTregs against heart allograft rejection in WT and Gulo-KO recipient mice

**Fig 4:**
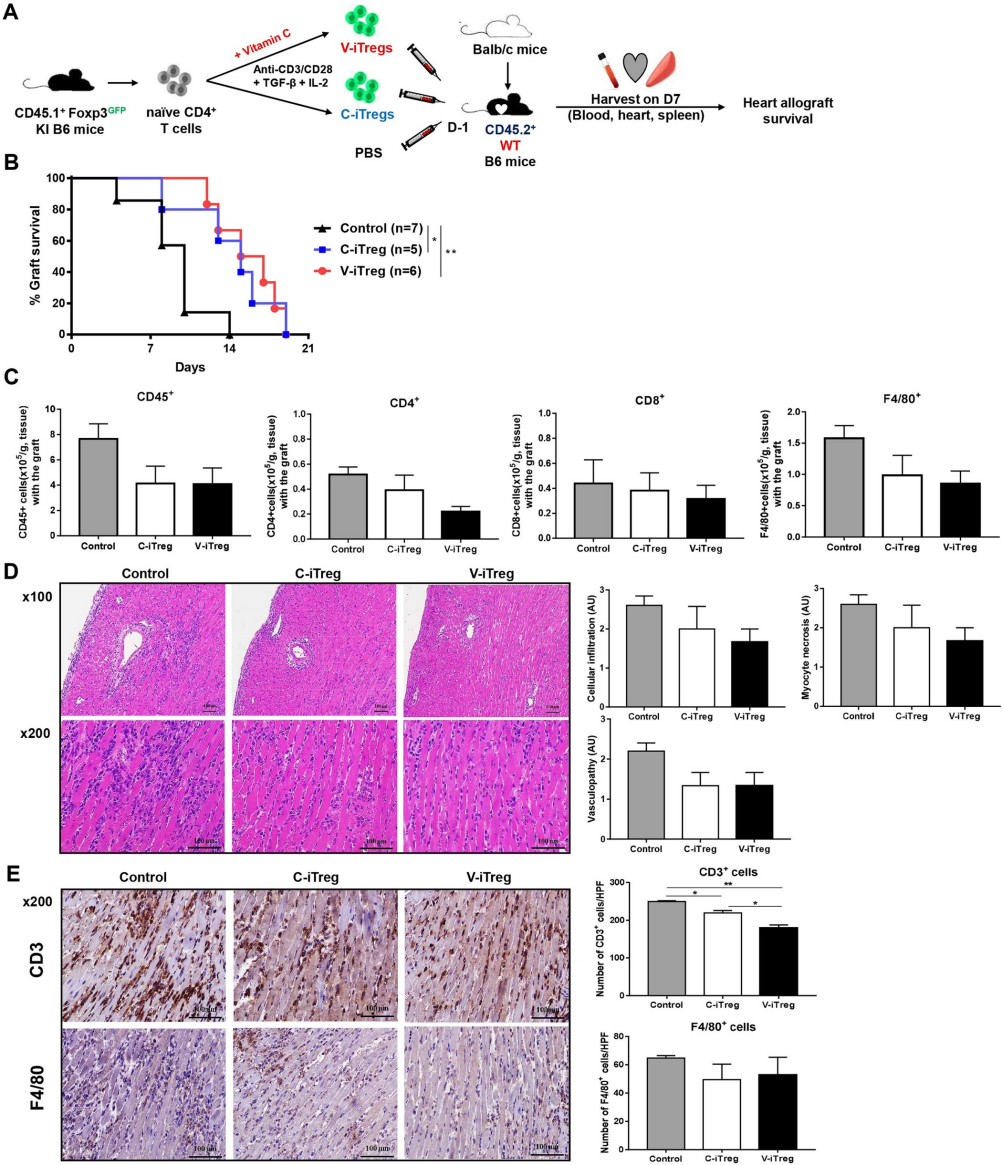
Impact of vitamin C on *in vivo* suppressive activity of iTregs against heart allograft rejection in WT mice. (A) Either V-iTregs or C-iTregs from WT mice were transferred to WT mice one day before transplantation and hearts from BALB/c mice were transplanted into B6 mice. (B) Heart allograft survival rates in the V-iTreg group were compared with those in the C-iTreg group and the control group without iTreg transfer. *P < 0.05, **P < 0.01 by log-rank test. (C) Flow cytometric analysis of inflammatory cell infiltration into heart allografts on day 7 (CD45^+^ leukocytes, CD4^+^ T cells, CD8^+^ T cells, and F4/80^+^ macrophages). N = 5–8 per group. Line and whiskers in the dot plots indicate the mean and standard error of the mean. (D) H & E staining results for heart allografts on day 7. Tissue injury was semi-quantitatively assessed regarding cellular infiltration, myocyte necrosis, and vasculopathy. N = 3–5 per group. Line and whiskers in the dot plots indicate the mean and standard error of the mean. Magnification, 100× or 200×. (E) Immunohistochemical staining results for CD3^+^ T cells and F4/80^+^ macrophages in heart allografts on day 7. N = 3 per group. Line and whiskers in the dot plots indicate the mean and standard error of the mean. *P < 0.05, **P < 0.01 by Student’s *t*-test. Magnification, 200×. Histologic images are representative of each group. C-iTregs, control induced regulatory T cells without vitamin C treatment; Foxp3, Forkhead Box P3; GFP, green fluorescent protein; H&E, hematoxylin and eosin; IL-2, interleukin-2; KI, knockin; PBS, phosphate-buffered saline; TGF-β, transforming growth factor-β; V-iTregs, vitamin C-treated, induced regulatory T cells; WT, wild type.

Adoptive transfer of either C- or V-iTregs prolonged heart allograft survival in WT mice. However, there was no significant difference between the C- and V-iTreg groups (Fig 4A and 4B). Flow cytometric analysis on day 7 demonstrated that V-iTregs as well as C-iTregs showed a trend toward suppressing allograft infiltration of CD45^+^ leukocytes, CD4^+^ T cells, CD8^+^ T cells, and F4/80^+^ macrophages compared with that of the control group without iTreg transfer, albeit without statistical significance (Fig 4C). Histologic tissue analysis on day 7 showed a trend of less injury in the V-iTreg and the C-iTreg groups than in control mice (Fig 4D). Immunohistochemical analysis on day 7 also showed a trend in perigraft infiltration of CD3^+^ T cells and F4/80^+^ macrophages, similar to flow cytometry data (Fig 4E).

**Fig 5:**
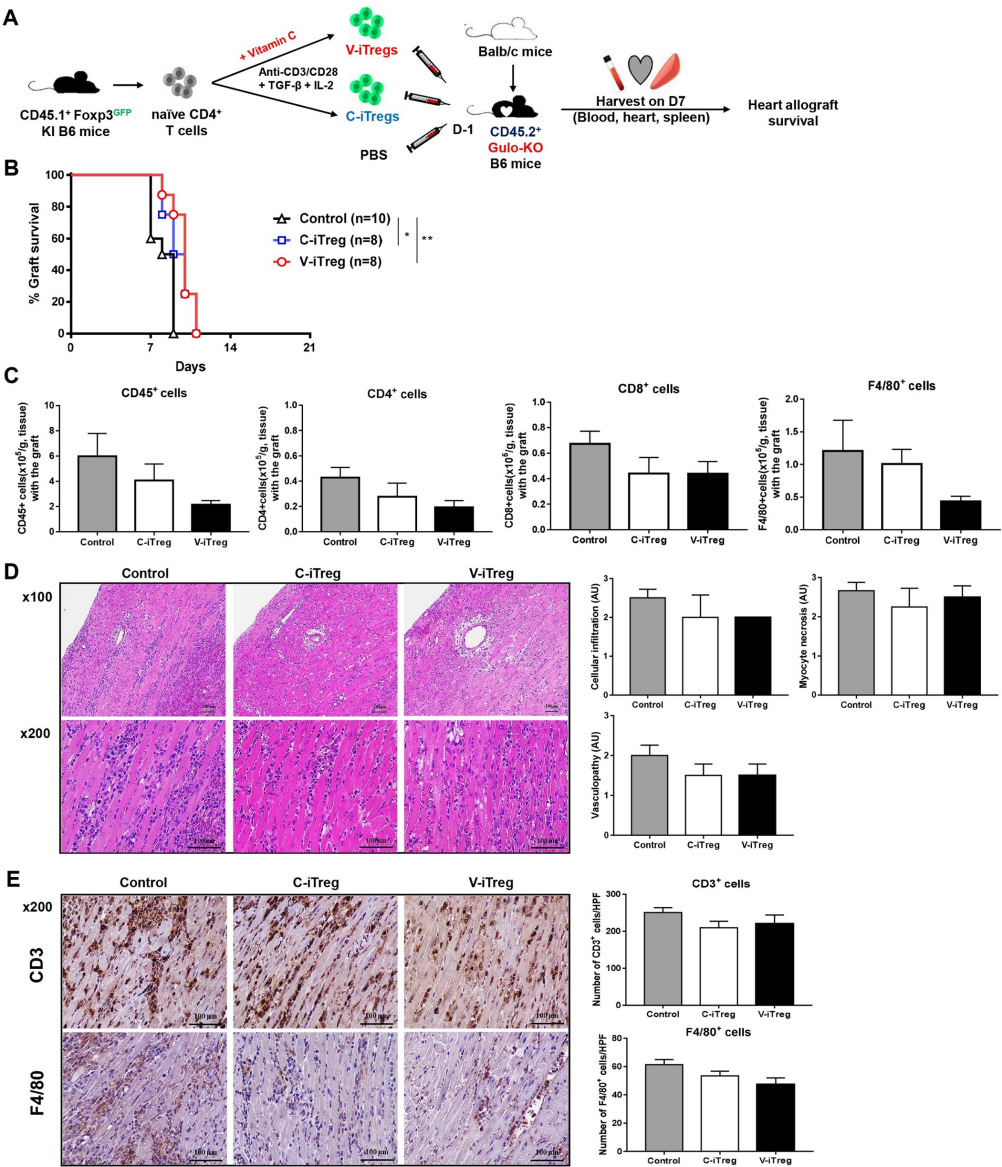
Impact of vitamin C on *in vivo* suppressive activity of iTregs against heart allograft rejection in Gulo-KO mice. (A) Either V-iTregs or C-iTregs from WT mice were transferred to Gulo-KO mice one day before transplantation and hearts from BABL/c mice were transplanted to Gulo-KO B6 mice. (B) Heart allograft survival rates in the V-iTreg group were compared with those in the C-iTreg group and the control group without iTreg transfer. *P < 0.05, **P < 0.01 by log-rank test. (C) Flow cytometric analysis of inflammatory cell infiltration into heart allograft on day 7 (CD45^+^ leukocytes, CD4^+^ T cells, CD8^+^ T cells, and F4/80^+^ macrophages). N = 5–6 per group. Line and whiskers in the dot plots indicate the mean and standard error of the mean. (D) H & E staining results for heart allografts on day 7. Tissue injury was semi-quantitatively assessed with regard to cellular infiltration, myocyte necrosis, and vasculopathy. N = 4–6 per group. Line and whiskers in the dot plots indicate the mean and standard error of the mean. Magnification, 100× or 200×. (E) Immunohistochemical staining results for CD3^+^ T cells and F4/80^+^ macrophages in heart allografts on day 7. N = 3 per group. Line and whiskers in the dot plots indicate the mean and standard error of the mean. Magnification, 200×. Histological images are representative of each group. C-iTregs, the control induced-regulatory T cells without vitamin C treatment; Foxp3, Forkhead Box P3; GFP, green fluorescent protein; Gulo-KO, L-gulonolactone-γ-oxidase knockout; H&E, hematoxylin and eosin; IL-2, interleukin-2; KI, knockin; PBS, phosphate-buffered saline; TGF-β, transforming growth factor-β; V-iTregs, vitamin C-treated, induced regulatory T cells; WT, wild type.

When iTregs were transferred to Gulo-KO mice one day before heart transplantation, both C- and V-iTreg prolonged heart allograft survival to a similar extent (Fig 5A and 5B). Flow cytometric analysis on day 7 showed a trend toward less inflammatory cell infiltration into the heart allograft in both V-iTreg and C-iTreg groups as compared to the control group without iTreg transfer (Fig 5C). Histologic tissue injury by rejection on day 7 showed a trend of less injury in the V-iTreg and the C-iTreg groups than in the control group (Fig 5D). Immunohistochemical study on day 7 also showed a similar trend in perigraft infiltration of CD3^+^ T cells and F4/80^+^ macrophages, as in flow cytometry data (Fig 5E). Concentrations of vitamin C (mean ± SEM) in the WT and the Gulo-KO mice were 21.45 ± 0.48 μg/mL and 3.52 ± 0.03 μg/mL, respectively (S2 Fig). Taken together, the *in vivo* suppressive activity of V-iTregs against heart allograft rejection was similar to that of C-iTregs in both WT and Gulo-KO recipient mice.

**Fig 6:**
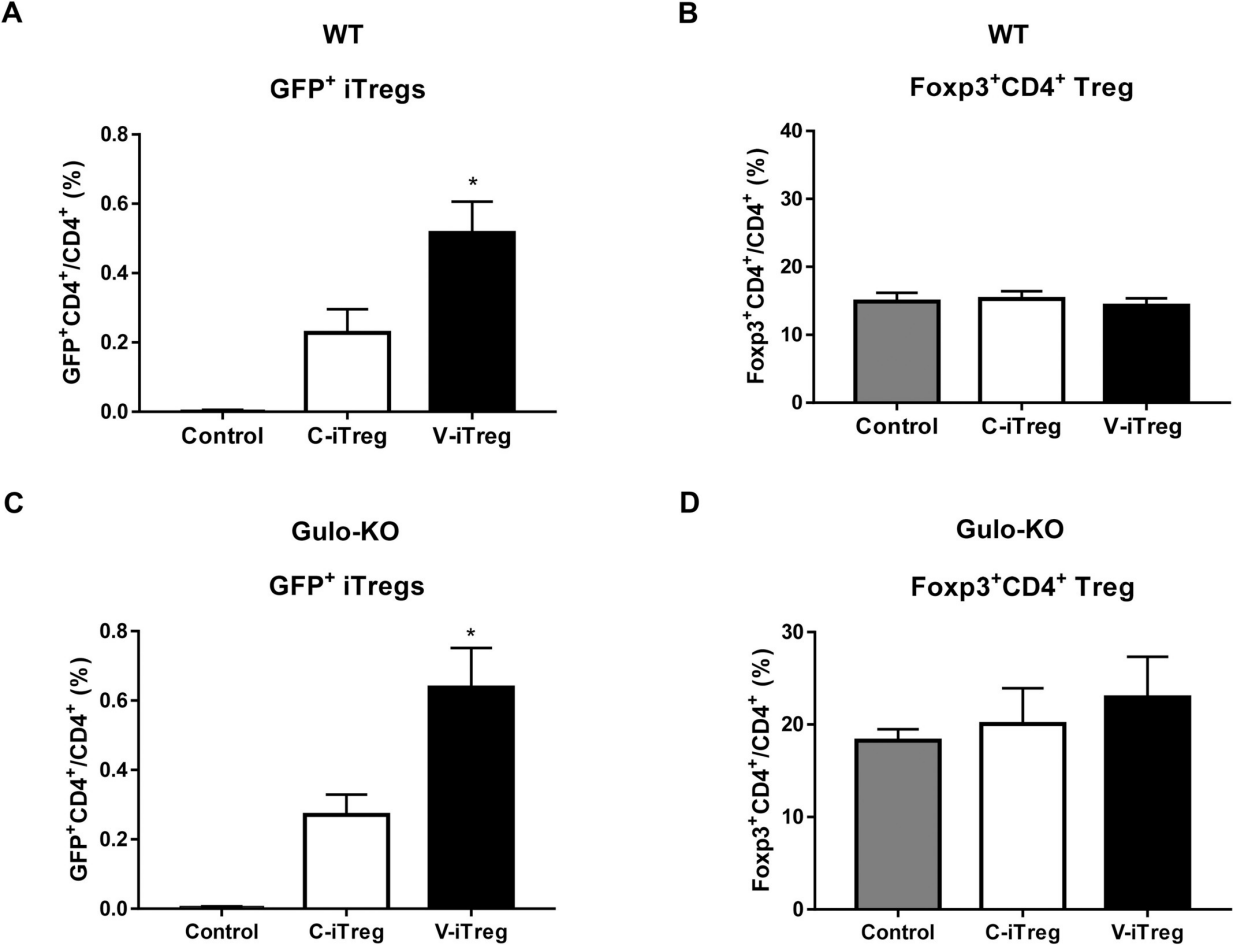
Impact of vitamin C on Foxp3 stability of V-iTregs and C-iTregs after heart transplantation in both WT and Gulo-KO mice. (A) Proportion of Foxp3^+^ among transferred iTregs (GFP^+^CD4^+^) in the spleens of WT mice on day 7 following heart transplantation. (B) Proportion of total Foxp3^+^CD4^+^ Tregs in the spleens of WT mice on day 7 following heart transplantation. (C) Proportion of Foxp3^+^ among transferred iTregs (GFP^+^CD4^+^) in the spleens of Gulo-KO mice on day 7 following heart transplantation. (D) Proportion of total Foxp3^+^CD4^+^ Tregs in the spleens of Gulo-KO mice on day 7 following heart transplantation. Line and whiskers in the dot plots indicate the mean and SEM, respectively. *P < 0.05 compared to the control group by Student’s *t*-test. C-iTregs, the control induced-regulatory T cells without vitamin C treatment; Foxp3, Forkhead Box P3; GFP, green fluorescent protein; Gulo-KO, L-gulonolactone-γ-oxidase knockout; V-iTregs, vitamin C-treated, induced regulatory T cells; WT, wild type.

Next, we analyzed transferred iTregs (GFP^+^CD4^+^) in the spleens of WT mice 1 week after heart transplantation. V-iTregs were more prevalent than C-iTregs (Fig 6A), whereas there was no difference in the overall Foxp3^+^CD4^+^ Tregs (Fig 6B). Similarly, there were more transferred GFP^+^ V-iTregs than GFP^+^ C-iTregs in the spleens on day 7 after heart transplantation in Gulo-KO mice (Fig 6C) although there was no difference in the overall Foxp3^+^CD4^+^ Tregs (Fig 6D). These data suggest that V-iTregs may possess better *in vivo* Foxp3 stability than C-iTregs in both WT and Gulo-KO mice.

### Role of vitamin C supplementation in V-iTreg-mediated suppression of allograft rejection

**Fig 7:**
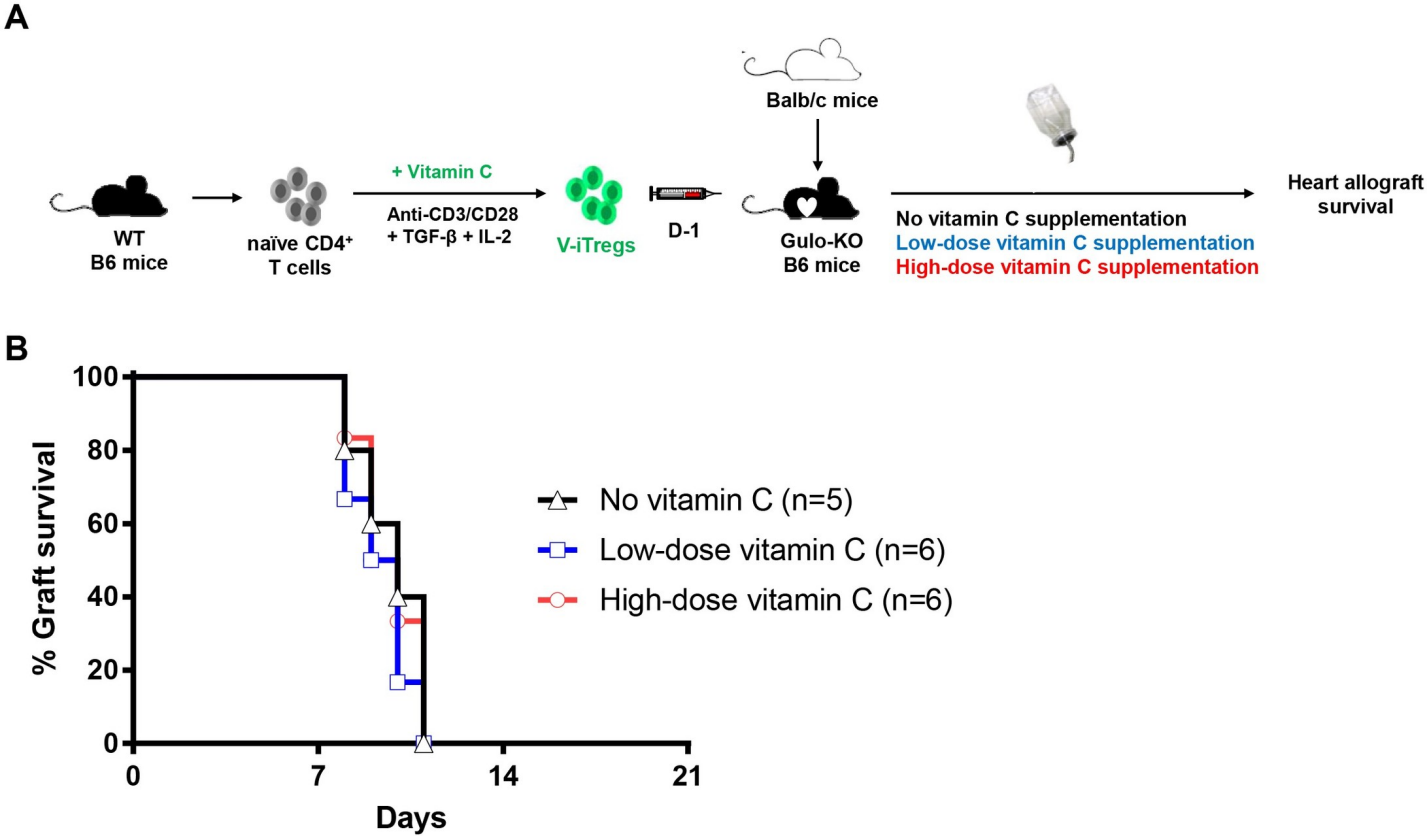
Role of vitamin C supplementation in V-iTreg-mediated suppression against allograft rejection. (A) V-iTregs from WT B6 mice were transferred to Gulo-KO B6 mice one day before BALB/c heart transplantation. Then, low- or high-dose vitamin C was administered to recipient mice via drinking water. (B) Heart allograft survival rates in no vitamin C group, low-dose vitamin C supplementation group, and high-dose vitamin C supplementation group. Gulo-KO, L-gulonolactone-γ-oxidase knockout; IL-2, interleukin-2; KI, knockin; TGF-β, transforming growth factor-β; V-iTregs, vitamin C-treated, induced regulatory T cells.

Next, we investigated whether supplementation with low- or high-dose vitamin C would improve V-iTreg-mediated suppression of heart allograft rejection in Gulo-KO mice (Fig 7A). Concentration of vitamin C in the low-dose supplementation group was 4.05 ± 0.08 μg/mL, which was similar to that in the no supplementation group (S2 Fig). However, concentration of vitamin C in the high-dose supplementation group (20.65 ± 0.28 μg/mL) was higher than the no supplementation group but similar to that in the WT mice (S2 Fig). When heart allograft survival rates were compared, neither low- nor high-dose vitamin C supplementation improved heart allograft survival in Gulo-KO recipients that had received V-iTregs (Fig 7B). These data demonstrated that further vitamin C supplementation did not improve the immunosuppressive activity of V-iTregs against heart allograft survival irrespective of dose.

## Discussion

The results of the current study indicated that V-iTregs from WT or Gulo-KO mice displayed better *in vitro* stability and suppressive activity than C-iTregs. V-iTregs also showed better *in vivo* stability than C-iTregs in both WT and Gulo-KO mice. However, V-iTregs did not show better suppressive activity against heart allograft rejection compared to C-iTregs in either WT or Gulo-KO mice. Moreover, supplementation with low- or high-dose vitamin C did not induce a significant improvement in heart allograft survival in Gulo-KO mice that had received V-iTregs.

CD4^+^ T cells express glucose transporters (GLUTs)-1, GLUT-3, and sodium-dependent vitamin C transport (SVCT2)-2. Foxp3^+^CD4^+^ Tregs have a higher expression of SVCT-2 than CD4^+^ non-Tregs [25]. Uptake of vitamin C by T cells enables modulation of T cell responses [25,26]. Vitamin C has previously been shown to improve *in vitro* and *in vivo* Foxp3 stability via epigenetic regulation [15,16]. In parallel, this study also showed that V-iTregs from WT mice showed better *in vitro* and *in vivo* Foxp3 stability than C-iTregs. Moreover, while the *in vitro* and *in vivo* Foxp3 stability of V-iTregs was better than that of C-iTregs in Gulo-KO mice, it was largely similar in extent to that in WT mice. Interestingly, vitamin C supplementation in the conversion phase of iTregs was beneficial for Foxp3 stability. However, once iTregs were generated, further supplementation of vitamin C to iTregs from either WT or Gulo-KO mice did not improve the Foxp3 stability of V-iTregs.

Consistent with the results of previous studies [15,16], V-iTregs from WT mice showed slightly better *in vitro* suppressive activity than C-iTregs. Furthermore, V-iTregs from Gulo-KO mice had slightly better *in vitro* suppressive activity than C-iTregs from Gulo-KO mice. These results could be attributed to similarities in the effects of vitamin C on Foxp3 stability of V-iTregs in Gulo-KO mice and WT mice.

Previous studies showed that V-iTregs prolonged skin allograft survival to a greater extent than did C-iTregs, which is consistent with their better *in vitro* suppressive activity. However, these studies had induced V-iTregs with vitamin C together with vitamin A or sodium butyrate [19,27]. On the other hand, systemic administration of vitamin C alone did not improve skin allograft survival in vitamin C-sufficient RAG KO mice, although vitamin C increased generation of iTregs and Foxp3 levels [25]. We found that adoptive transfer of V-iTregs prolonged heart allograft survival in WT mice, as C-iTregs prolonged heart allograft rejection in a previous study [28]; however, there was no significant difference in *in vivo* suppressive activities against heart allograft rejection between V-iTregs and C-iTregs. These results suggest that vitamin C alone may not be sufficient to stabilize iTregs and improve their suppressive activity against allograft rejection, a very stringent immune response. We also investigated whether V-iTregs displayed better suppressive activity against heart allograft rejection in Gulo-KO mice, which mimic humans, as they lack gulo. In Gulo-KO mice, V-iTregs did not show better *in vivo* suppressive activity against heart allograft rejection than C-iTregs. Overall, the immunosuppressive effects of V-iTregs on alloimmune responses were not better than those of C-iTregs in either WT or Gulo-KO mice.

Next, we investigated whether *in vivo* vitamin C supplementation would improve immunosuppressive activity of V-iTregs against heart allograft rejection in Gulo-KO recipients, as also the optimal dose of vitamin C supplementation. Supplementation with neither low- nor high-dose Vitamin C induced a significant change in heart allograft survival in Gulo-KO recipients that had received V-iTregs. These results suggest that vitamin C body content does not have a significant impact on the immunosuppressive activity of V-iTregs against allograft rejection, once V-iTregs are successfully generated in the presence of vitamin C. These *in vivo* results are consistent with the *in vitro* findings that vitamin C supplementation after generation of iTregs was not helpful for the maintenance of Foxp3 in iTregs.

A limitation associated with the current study was that we did not combine vitamin C with other agents, such as vitamin A and histone modifiers, for stabilization of Foxp3 in iTregs to boost the beneficial effects of vitamin C on iTregs [16,27,29,30]. P2X7 receptor blockade suppressed effector T cells or induced Tregs, thereby suppressing ischemia-reperfusion injury and allograft rejection [31,32]. Therefore, combination of V-iTregs with P2X7 receptor blockade would augment the suppressive effects of V-iTregs on allograft rejection. However, the main focus of this study was to elucidate the role of vitamin C in various aspects of iTregs from generation to immunosuppressive actions in vitamin C-deficient conditions compared to vitamin C–sufficient conditions. Further studies are warranted to address the role of iTregs that were generated in the presence of vitamin C along with other agents, as well as combinatory effects of V-iTregs and other immune modulatory agents.

Nevertheless, this study elucidated the role of vitamin C in the generation, maintenance, and immunosuppressive activity of iTregs in vitamin C-deficient conditions that may be relevant to human conditions, as well as vitamin C-sufficient conditions. Furthermore, we demonstrated similar *in vivo* immunosuppressive activity of V-iTregs against allograft rejection with that of C-iTregs using a real vascularized organ transplant model instead of a skin transplant model in Gulo-KO mice as well as in WT mice.

In conclusion, administration of vitamin C in the generation phase of iTregs improved Foxp3 stability of iTregs under both vitamin C-sufficient and -deficient conditions. However, V-iTregs do not show better immunosuppressive activity against heart allograft rejection compared to C-iTregs under either vitamin C-sufficient or -deficient conditions.

## Supporting information

S1 Fig Comparison of nTregs between WT and Gulo-KO mice. Proportions of Foxp3^+^CD4^+^ nTregs among CD4^+^ T cells (A) and Foxp3 MFI of nTregs (B) in the Gulo-KO mice were compared with those in WT mice. Foxp3, Forkhead Box P3; Gulo-KO, L-gulonolactone-γ-oxidase knockout; MFI, mean fluorescence intensity; nTregs, naturally occurring regulatory T cells; WT, wild type.
(TIF)

S2 Fig Concentrations of vitamin C. Serum vitamin C concentrations were measured at 9 to 11 d after heart transplantation in WT, Gulo-KO mice without vitamin C supplementation, and Gulo-KO mice with low- or high-dose vitamin C supplementation. Gulo-KO, L-gulonolactone-γ-oxidase knockout; WT, wild type. **P < 0.01 compared to the WT mice group; ^##^P < 0.05, compared to the Gulo-KO mice group without vitamin C supplementation by Student’s *t*-test.
(TIF)

S1 Table List of antibodies used in the study. (DOCX)

S1 Appendix Experimental protocol of mouse heart transplantation. (DOCX)
