# Supplementary figures and images for: Suppressive effects of vitamin C-treated induced-regulatory T cells on heart allograft rejection under vitamin C-deficient or –sufficient conditions
Source: PLoS One. 2021 Feb 12;16(2):e0246967. doi: 10.1371/journal.pone.0246967 (PMC7880463; doi:10.1371/journal.pone.0246967)

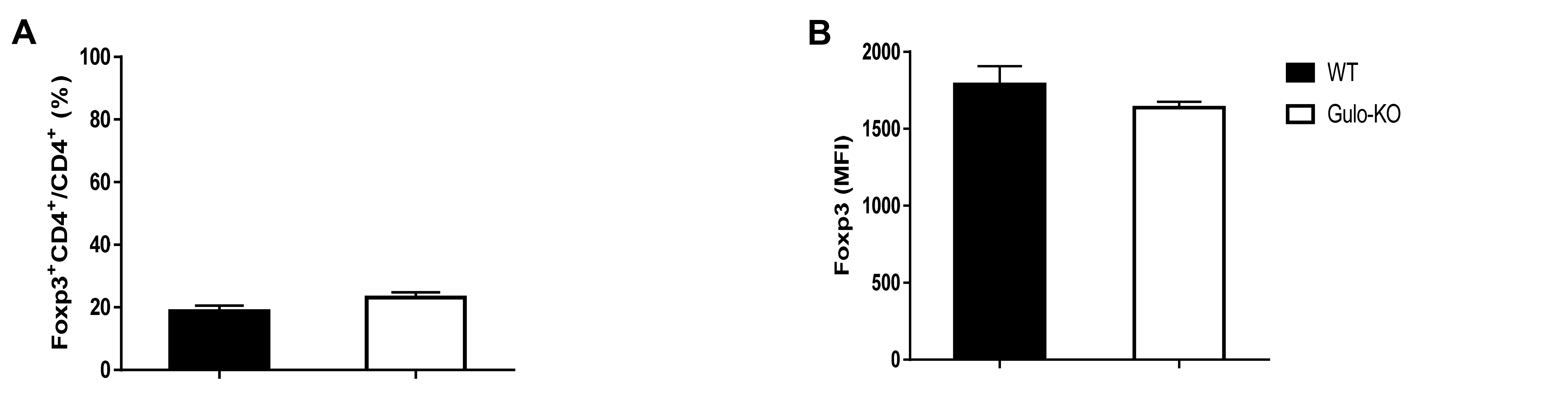

Supplement: S1 Fig — Proportions of Foxp3+CD4+ nTregs among CD4+ T cells (A) and Foxp3 MFI of nTregs (B) in the Gulo-KO mice were compared with those in WT mice. Foxp3, Forkhead Box P3; Gulo-KO, L-gulonolactone-γ-oxidase knockout; MFI, mean fluorescence intensity; nTregs, naturally occurring regulatory T cells; WT, wild type. (TIF) [file pone.0246967.s001.tif]

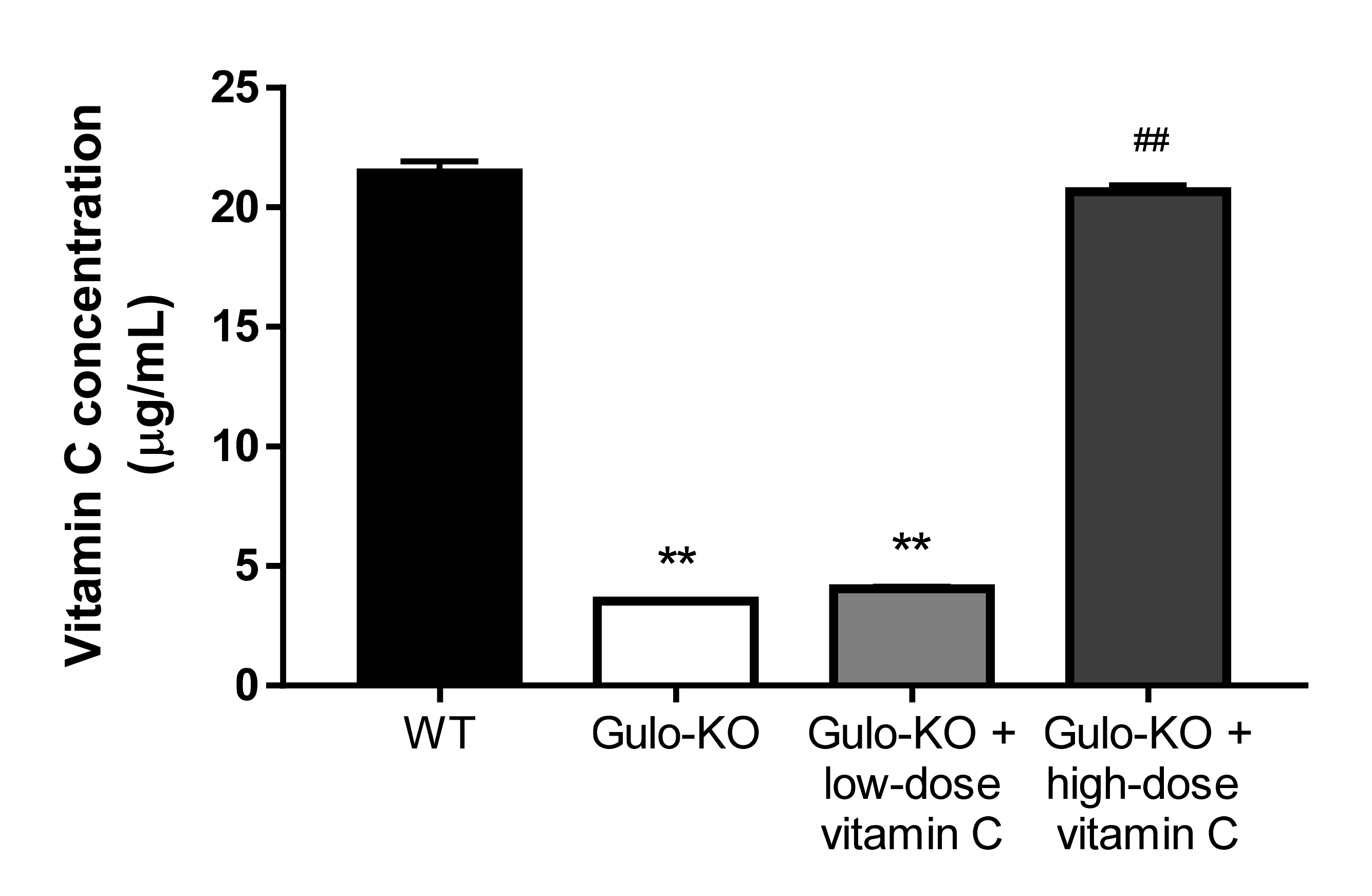

Supplement: S2 Fig — Serum vitamin C concentrations were measured at 9 to 11 d after heart transplantation in WT, Gulo-KO mice without vitamin C supplementation, and Gulo-KO mice with low- or high-dose vitamin C supplementation. Gulo-KO, L-gulonolactone-γ-oxidase knockout; WT, wild type. **P < 0.01 compared to the WT mice group; ##P < 0.05, compared to the Gulo-KO mice group without vitamin C supplementation by Student’s t-test. (TIF) [file pone.0246967.s002.tif]
