# Supplementary material for: Suppressive effects of vitamin C-treated induced-regulatory T cells on heart allograft rejection under vitamin C-deficient or –sufficient conditions
Source: PLoS One. 2021 Feb 12;16(2):e0246967. doi: 10.1371/journal.pone.0246967 (PMC7880463; doi:10.1371/journal.pone.0246967)
Supplement: S1 Table — (DOCX) [file pone.0246967.s003.docx]

**S1 Table 1. Lists of antibodies used in the study.**

| Target | Host | Reactivity | Clone | Conjugated fluorochrome | Use | Reaction concentration | Catalogue number | Company information |
| --- | --- | --- | --- | --- | --- | --- | --- | --- |
| B220 | Rat | Mouse, Human | RA3-6B2 | APC | FACS | 2 μg/mL | 553092 | BD Biosciences |
| CD11c | Armenian Hamster | Mouse | N418 | PE | FACS | 2 μg/mL | 565592 | BD Biosciences |
| CD25 | Rat | Mouse | PC61 | PE-Cy7 | FACS | 2 μg/mL | 552880 | BD Biosciences |
| CD3 | Rat | Mouse | 17A2 | APC | FACS | 2 μg/mL | 100235 | BioLegend |
| CD4 | Rat | Mouse | GK1.5 | PE-Cyanine7 | FACS | 2 μg/mL | 25-0041-82 | Thermo Fisher Scientific |
| CD44 | Rat | Mouse | IM7 | APC | FACS | 2 μg/mL | 559250 | BD Biosciences |
| CD45 | Rat | Mouse | 30-F11 | Brilliant Violet 421 | FACS | 2 μg/mL | 103133 | BioLegend |
| CD45.1 | Mouse | Mouse | A20 | APC/Cyanine7 | FACS | 2 μg/mL | 110715 | BioLegend |
| CD45.2 | Mouse | Mouse | 104 | APC | FACS | 2 μg/mL | 109813 | BioLegend |
| CD8a | Rat | Mouse | 53-6.7 | PE | FACS | 2 μg/mL | 561095 | BD Biosciences |
| F4/80 | Rat | Mouse | BM8 | PE | FACS | 2 μg/mL | 12-4801-82 | Thermo Fisher Scientific |
| Foxp3 | Rat | Mouse, Rat | FJK-16S | FITC | FACS | 5 μg/mL | 11-5773-82 | Thermo Fisher Scientific |
| CD3 | Domestic Rabbit | Mouse, Human | - | Unconjugated | IHC | 0.25 | A0452 | DAKO |
| F4/80 | Rat | Mouse | BM8 | Unconjugated | IHC | 0.25 | 14-4801-82 | Thermo Fisher Scientific |

APC, allophycocyanin; CD, cluster of differentiation; FACS, fluorescence-activated cell sorting; FITC, fluorescein isothiocyanate; IHC, immunohistochemistry; PE, phycoerythrin; PE-Cy7, phycoerythrin cyanine7.
