# Supplementary material for: Suppressive effects of vitamin C-treated induced-regulatory T cells on heart allograft rejection under vitamin C-deficient or –sufficient conditions
Source: PLoS One. 2021 Feb 12;16(2):e0246967. doi: 10.1371/journal.pone.0246967 (PMC7880463; doi:10.1371/journal.pone.0246967)
Supplement: S1 Appendix — (DOCX) [file pone.0246967.s004.docx]

**S1 Appendix**

**Experimental protocol of mouse heart Transplantation**

The heterotopic heart transplantation was performed using 8-week-old BABL/c mice weighing 25 to 27 g as donors; and WT or Gulo-KO C57BL/6 mice weighing 25 to 27 g as recipients. The mice were anesthetized by inhalation of 1-2% of isoflurane (Hana Pharm, Republic of Korea). The donor underwent a thoracotomy, by incising the chest cavity, to expose the heart and blood vessels. One mL of heparinized saline solution was injected into the inferior vena cava (IVC) to release the blood. The blood vessel was ligated with 7-0 silk suture, the aorta and pulmonary artery were exposed, and the donor heart was procured. In turn, the proximal abdominal aorta and IVC segments of recipients were clamped with small vessel clamps. End-to-side anastomosis of the donor aorta and the pulmonary artery to the abdominal aorta and IVC of the recipient was performed. After transplantation, the vascular clip was released for revascularization. Warm saline solution at 37 °C was spread on the graft to help recovery and 0.6 mL of normal saline solution was subcutaneously injected to prevent dehydration. Postoperatively, tramadol hydrochloride (0.25 mg/kg, Jeil Pharmaceutical, Seoul, Republic of Korea) was injected to relieve pain, and cefotaxime (100 mg/kg, Shinpoong, Seoul, Republic of Korea) was intraperitoneally administrated for 3 days to prevent infection. The recipient mice were cared in incubator chamber (Jeung Do Bio & Plant Co, Republic of Korea) at 27 °C for a day to maintain body temperature. Heart allograft rejection was monitored by the heartbeat of the graft assessed directly by abdominal palpation. The intensity of the beating was recorded and scored with values ranging from 4 (normal amplitude and frequency) to 0 (rejected graft without beating).
